# Importance in the Occurrence Distribution of Minimum Oropharyngeal Cross-Sectional Area in the Different Skeletal Patterns Using Cone-Beam Computed Tomography

**DOI:** 10.1155/2021/5585629

**Published:** 2021-05-05

**Authors:** Ying-Sheng Chen, Szu-Ting Chou, Jung-Hsuan Cheng, Shis-Chieh Chen, Chin-Yun Pan, Yu-Chuan Tseng

**Affiliations:** ^1^Dental Department, Taipei Medical University-Shuang Ho Hospital, Taipei, Taiwan; ^2^Department of Orthodontics, Kaohsiung Medical University Hospital, Kaohsiung, Taiwan; ^3^School of Dentistry, College of Dental Medicine, Kaohsiung Medical University, Kaohsiung, Taiwan

## Abstract

**Purpose:**

Obstructive sleep apnea is a condition involving repetitive partial or complete collapse of the pharyngeal airway, especially in patient with mandibular hypoplasia. The present study investigated the differences between the volume of the oropharyngeal airway and the minimum axial area in three skeletal patterns through the use of cone-beam computed tomography (CBCT).

**Materials and Methods:**

CBCT scans of 147 patients were collected to measure the upper oropharyngeal airway volume (UOV), lower oropharyngeal airway volume (LOV), upper oropharyngeal airway area (UOA), minimum upper oropharyngeal airway area (MUOA), lower oropharyngeal airway area (LOA), minimum lower oropharyngeal airway area (MLOA), anatomical structures (orbitale, Or; porion, Po; pogonion, Pog; hyoid, H; second cervical vertebra, C2; fourth cervical vertebra, C4), and relevant angles. Statistical analysis was performed using analysis of variance and Pearson's test.

**Results:**

Compared with patients in Class II, those in Class III and Class I exhibited a significantly anterior position of H and Pog. The vertical positions of H and Pog revealed no significant difference between the three skeletal patterns. Patients in skeletal Class III exhibited significantly larger oropharyngeal area (UOA, MUOA, LOA, MLOA) and oropharyngeal airway (UOV and LOV) than those in skeletal Class II did. The horizontal position of Pog had a moderately significant correlation with UOA (*r* = 0.471) and MUOA (*r* = 0.455).

**Conclusion:**

Patients in skeletal Class II had significantly smaller oropharyngeal airway areas and volumes than those in Class III did. The minimum oropharyngeal cross-sectional area had a 67% probability of occurrence in the upper oropharyngeal airway among patients in Class I and Class II and a 50% probability of occurrence among patients in Class III.

## 1. Introduction

The pharynx is a conical channel linking the oral and nasal cavity to the esophagus and trachea. It is located at the intersection of the digestive and respiratory tracts and serves as the passage for food and air. Thus, the pharynx plays a crucial role in swallowing and breathing functions [1, 2]. The pharyngeal airway is divided into three parts, namely, the nasopharynx, oropharynx, and laryngopharynx. The nasopharynx and the oropharynx are demarcated by the soft palate to the rear of the palate, and the oropharynx and laryngopharynx are demarcated by the apex of the epiglottis. The structure of the pharynx affects the volume of the respiratory tract, facial growth pattern, masticatory pattern, and the risk of obstructive sleep apnea. The anatomical structure of the pharyngeal airway space varies according to the diverse growth patterns of the maxilla and mandible.

Face-driven orthodontics and mandibular setback surgery can cause the backward movement of teeth, leading to changes in the pharyngeal airway space [3]. Thus, evaluating and measuring the pharyngeal airway space of patients are important before orthodontic treatment and orthognathic surgery. Such precautions can avoid the backward movement of teeth and prevent the mandible from pushing the tongue further backward, which ultimately oppresses and reduces the pharyngeal airway space, causing obstructive sleep apnea in more serious cases. Compared with the nasopharyngeal and laryngopharyngeal airways, the oropharyngeal airway is more likely to be influenced by the surrounding organs. The dimensions of the oropharyngeal airway are mainly affected by the anteriority or posteriority of the mandibular position and tongue size. The front and rim of the tongue are attached to the mandible, and the base of the tongue is linked to the hyoid bone; connections also exist between the tongue and the soft palate as well as the palatoglossus muscles.

In the present study, cone-beam computed tomography (CBCT) was used to explore differences in the volume and minimum cross-sectional area of the individual parts of the oropharyngeal airway in terms of skeletal patterns. In addition, this study involved evaluation of the relationships between the maxilla and mandible; the relationships between sex, age, and the cervical spine; other anatomical structures (including the mandible and hyoid bone positions); the related distances or angles (head and cervical spine positions) that might affect oropharyngeal airway dimensions; and relationships between oropharyngeal airway volume and the minimum cross-sectional area.

## 2. Materials and Methods

The CBCT scans (New Tom VGi evo, Imola, Italy) of 147 patients were collected from the dental department of Kaohsiung Medical University Chong-Ho Memorial Hospital. Patients with craniofacial disorders or malformation, those with pharyngeal or laryngeal pathology, and those with craniofacial injuries were excluded from the study. The patient characteristics included age, ANB angle, and body mass index (BMI). For analysis, the patients were divided into three groups according to their skeletal pattern: 30 patients (19 female and 11 male) in Class I (0° ≤ ANB ≤ 4°; average age: 25.3 years), 40 patients (28 female and 12 male) in Class II (ANB > 4°; average age: 25.8 years), and 77 patients (44 female and 33 male) in Class III (ANB < 0°; average age: 23.8 years).

**Figure 1:**
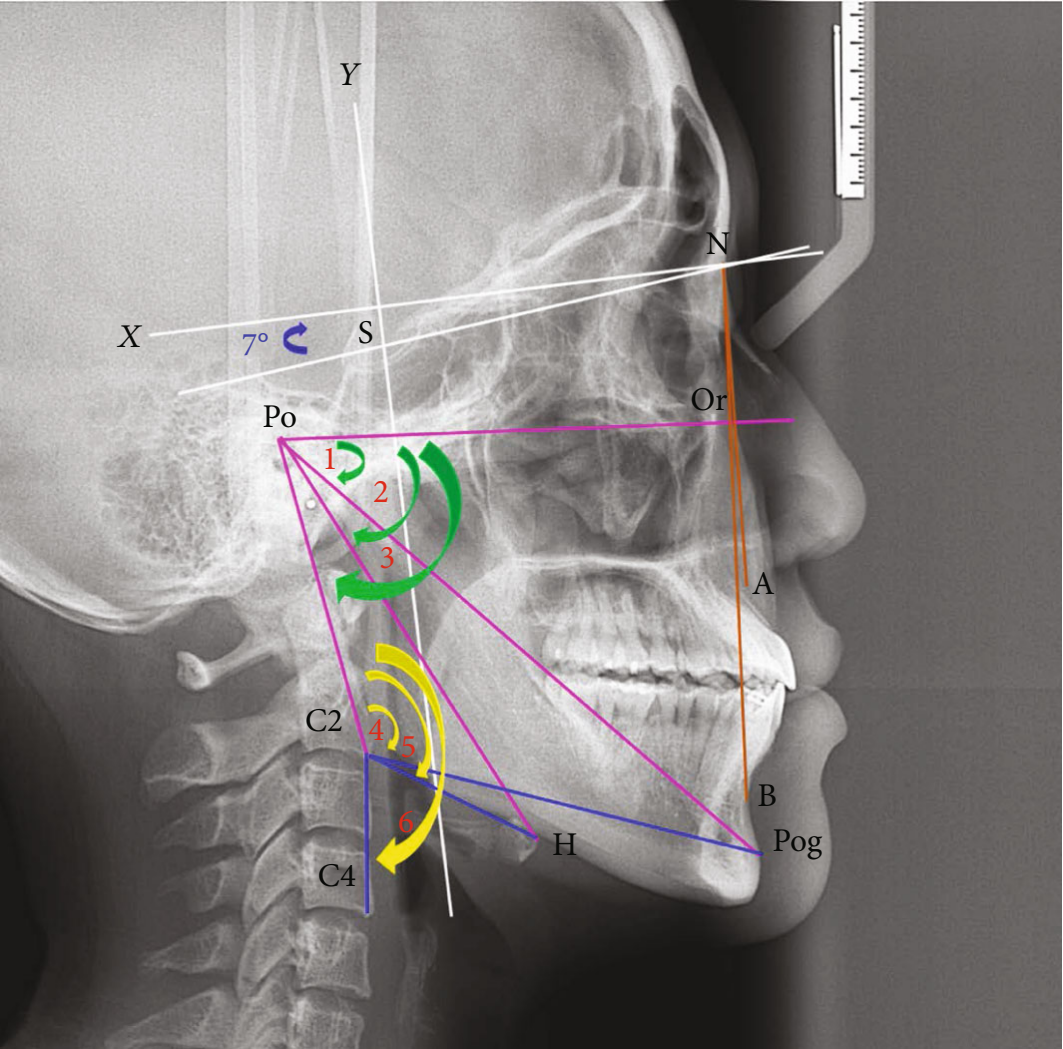
Landmarks: sella (S), nasion (N), A point (A), B point (B), pogonion (Pog), hyoid bone (H), second cervical vertebra (C2), and fourth cervical vertebra (C4). *X*-axis (white line): constructed by drawing a line through nasion 7° up from SN line. *Y*-axis (white line): a line through sella (S) perpendicular to the *X*-axis. The measured angles: brown color, ANB angle; green color, (1) Or–Po–Pog angle, (2) Or–Po–H angle, (3) Or–Po–C2 angle; and yellow color, (4) Po-C2-Pog angle, (5) Po-C2-H angle, (6) Po–C2–C4 angle.

CBCT images were imported using the Digital Imaging and Communications in Medicine into Dolphin® 11.0 software (Dolphin Imaging and Management Solutions, Chatsworth, CA, USA). The reference points (Figure 1) included sella (S), nasion (N), A point (A), B point (B), pogonion (Pog), the most superior and anterior point of the hyoid bone (H), tip at the end of the uvula (U), upper tip at the end of the epiglottis (E), inferoanterior point on the second cervical vertebra (C2), and inferoanterior point on the fourth cervical vertebra (C4). The coordinate system consisted of the *X*-axis (constructed by drawing a line through nasion 7° up from the SN line) and *Y*-axis (constructed by drawing a line through the S point perpendicular to the *X*-axis) [4]. The horizontal and vertical positions of H and Pog were investigated. The related angles were measured and included the head positions (Or–Po–Pog angle, Or–Po–H angle, and Or–Po–C2 angle) and cervical spine positions (Po-C2-Pog angle, Po-C2-H angle, and Po–C2–C4 angle).

**Figure 2:**
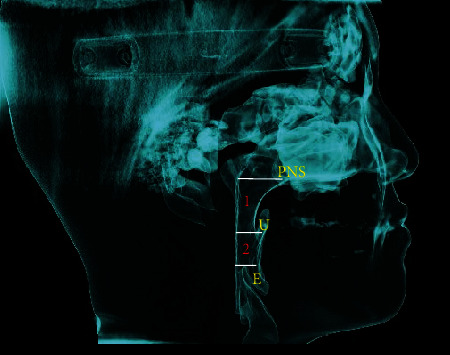
Landmarks: posterior nasal spine (PNS), uvula (U), epiglottis (E). The measured pharyngeal airway volume: (1) upper oropharyngeal airway volume: UOV; (2) lower oropharyngeal airway volume: LOV.

As shown in Figure 2, the Frankfort horizontal (FH) plane was defined as the plane connecting the right orbitale (Or) and porion (Po) on both sides. The oropharyngeal airway space was divided into the upper oropharyngeal airway (velopharyngeal airway) and lower oropharyngeal airway (glossopharyngeal airway). In the upper oropharyngeal airway, the upper bound of the pharyngeal airway passes through the posterior nasal spine (PNS) and is parallel to the standard horizontal plane, and the lower bound passes through the tip at the end of the uvula and is parallel to the standard horizontal plane. In the lower oropharyngeal airway, the upper bound of the pharyngeal airway passes through the tip at the end of the uvula and is parallel to the standard horizontal plane, and the lower bound passes through the upper tip at the end of the epiglottis and is parallel to the standard horizontal plane.

**Figure 3:**
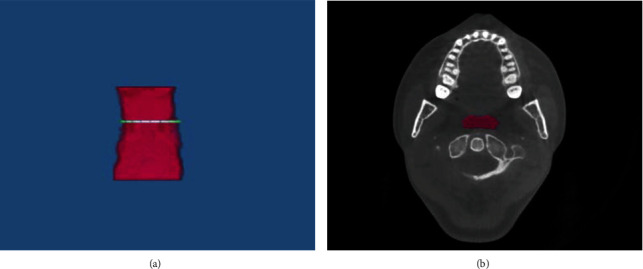
(a) In the 3D image, the minimum cross-sectional area (green color) of upper oropharyngeal airway in the oropharyngeal airway (pink color). (b) The minimum cross-sectional area (pink color) of upper oropharyngeal airway in the axial view.

In Figure 3, three-dimensional (3D) model of airway space was obtained by Dolphin® 3D software. Airway semiautomatic segmentation (including borders and landmarks) was defined as aforementioned. The airway volume and airway area were automatically calculated by the Dolphin® 3D software. The upper oropharyngeal airway volume (UOV), lower oropharyngeal airway volume (LOV), and total oropharyngeal airway volume (TOV = UOV + LOV) were measured. The oropharyngeal airway areas (axial view) were measured as follows: upper oropharyngeal airway area (UOA: passes through the tip at the end of the uvula), minimum upper oropharyngeal airway area (MUOA: the minimum cross-sectional area of UOV), lower oropharyngeal airway area (LOA: passes through the upper tip at the end of the epiglottis), minimum lower oropharyngeal airway area (MLOA: the minimum cross-sectional area of LOV), and minimum total oropharyngeal airway area (MTOA: the minimum cross-sectional area of TOV).

The present study investigated the differences between the various skeletal patterns in terms of the volume and area of the oropharyngeal airway. Statistical analysis was performed using SPSS (version 20; IBM, Armonk, NY, USA), and *p* < 0.05 was the criterion for statistical significance. The mean values among the groups were compared using one-way analysis of variance with post hoc Tukey HSD test. Pearson's correlation coefficient was used to compare the correlations among the variables of the groups. Regarding the absolute value of the correlation coefficient (*r*), 0–0.19 indicated a very weak correlation, 0.2–0.39 indicated a weak correlation, 0.40–0.59 indicated a moderate correlation, 0.6–0.79 indicated a strong correlation, and 0.8–1 indicated a very strong correlation. This study was approved by the Institutional Review Board of Kaohsiung Medical University Hospital (KMUHIRB-E(II)-20160066).

## 3. Results

No significant difference was observed between the patients in the three groups in terms of age or BMI (Table 1). In terms of the horizontal distance of H and Pog, that of patients in Class II (27.1 mm and 63.7 mm, respectively) was significantly smaller than that of patients in Class I (33.7 mm and 74.3 mm, respectively) and Class III (36.1 mm and 80.2 mm, respectively). No significant difference was observed between the groups with respect to the vertical position of H and Pog. The Or–Po–Pog and Or–Po–C2 angles (Table 2) of patients in Class II (48.9° and 87.3°, respectively) were significantly larger than those of patients in Class I (45.6° and 83.8°, respectively) and Class III (44.5° and 82.5°, respectively). The Po–C2–H angle of patients in Class I (130.6°) was significantly greater than that of patients in Class II (124.8°). The Po–C2–C4 angle of patients in Class III (189.4°) was significantly smaller than that of patients in Class I (192.7°) and II (193.2°).

Table 3 presents a comparison of oropharyngeal airway space in the three skeletal patterns. The UOA of patients in Classes III (468.5 mm^2^) and I (443.9 mm^2^) was significantly greater than that of patients in Class II (377.2 mm^2^). The MUOA (118.3 mm^2^), LOA (289.7 mm^2^), MLOA (113.4 mm^2^), UOV (13801.9 mm^3^), LOV (7773.5 mm^3^), MTOA (96 mm^2^), and TOV (21575.4 mm^3^) of patients in Class III were significantly greater than the corresponding values of patients in Class II (78.8 mm^2^, 225.4 mm^2^, 86.0 mm^2^, 10658.7 mm^3^, 6051.5 mm^3^, 69.6 mm^2^, and 16710.1 mm^3^, respectively). Evaluation of the distribution of MTOA revealed that 20 patients in Class I had MUOA and 10 had MLOA, 27 patients in Class II had MUOA and 13 had MLOA, and 39 patients in Class III had MUOA and 38 had MLOA. The MUOA represented the MTOA of the oropharyngeal airway in a two-thirds of patients in Class I (66.7%), a two-thirds of patients in Class II (67.5%), and one-half (50.6%) of patients in Class III.

On the basis of patient characteristics (Table 4), Pearson's test was performed to evaluate the correlations of pharyngeal airway space. Both the area and volume of each airway space were significantly positively correlated with sex: male patients had larger airways, indicating a positive correlation. Skeletal pattern had a significant positive correlation with MUOA and MTOA: the MUOA and MTOA of patients in Class III were larger, indicating a positive correlation. Age exhibited a significant negative correlation with MUOA, LOA, and MTOA; higher age was associated with a smaller MUOA, LOA, and MTOA. No significant correlation was observed between BMI and the area and volume of oropharyngeal airway space. Greater ANB angle was associated with a significantly smaller area and volume of oropharyngeal airway space. Except for PogY (vertical position) and MLOA, which were not significantly correlated, the positions of H and Pog were positively correlated with the areas and volumes of all of the other oropharyngeal airway spaces. The horizontal position of Pog was moderately correlated with UOA (*r* = 0.476) and MUOA (*r* = 0.455).

As shown in Table 5, the Or–Po–Pog angle was significantly negatively correlated (weak or very weak) with UOA, MUOA, UOV, LOA, TOV, and MLOA. The Or–Po–H angle was significantly positively correlated (weak or very weak) with MUOA, UOV, TOV, and LOV. None of the oropharyngeal airway spaces (areas and volumes) exhibited a significant correlation with the Or–PO–C2, Po–C2–Pog, or PO-C2-H angle. The Po–C2–C4 angle was significantly negatively correlated (weak or very weak) with UOA, MUOA, UOV, LOA, MTOA, and TOV.

## 4. Discussion

The volume of the pharyngeal airway can be affected by anatomical anomalies in both the soft tissue and craniofacial skeleton. According to functional matrix theory, proposed by Moss [5], the growth and development of the craniofacial area can be controlled by the functional activity of the soft tissue around the craniofacial skeleton. Thus, a direct interaction exists between the pharyngeal airway space and craniofacial morphology, and any anomaly in these spaces could affect the position of the surrounding bones. Related literature [6, 7] has reported rapid and ongoing growth of the pharyngeal structure before the age of 13 years that ceases between 14 and 18 years of age. On the basis of relevant research results, this study focused on the pharyngeal airways of patients aged over 16 years, which constitutes the most mature and stable period.

BMI is generally used to represent a patient's physical characteristics. In this study, no significant difference was observed between the age and BMI of the patients in the three groups, signifying similar demographic characteristics of all patients. Therefore, the results of this study were unaffected by differences in the physical characteristics of the patients, thereby revealing their actual oropharyngeal airway statuses with objective measurements. It is as expected that BMI exhibited no significant correlation with the area or volume of the oropharyngeal airway. Claudino et al. [8] and Tseng et al. [9] indicated that airway volume was significantly correlated with ANB angle, whereas Kula et al. [10] and Alves et al. [11] found no significant correlation between these elements. The current study confirmed the findings of Claudino et al. [8] and Tseng et al. [9] that the ANB angle is a crucial factor affecting airway dimensions. Alves et al. [11] reported significant differences between the airway volumes of male and female participants. However, a study by Solow et al. [12] revealed that sex did not significantly affect airway dimensions. The current study also noted a significant correlation between the oropharyngeal airway space and sex; the oropharyngeal airway space of male patients was larger than that of female patients.

El and Palomo [13] indicated that the relation between the position of the mandible and skull base also affects the oropharyngeal space. Kim et al. [14] indicated that compared with patients with normal skeletal anterior–posterior relationships, patients with a mandible positioned more to the rear had a smaller airway volume. Research reports [15, 16] have highlighted the crucial role of the hyoid bone and the muscle tissue attached to it in maintaining a normal airway space, and different positions of the mandible are often accompanied by diverse hyoid bone positions. Yamaoka et al. [16] revealed that the tongue base of patients with skeletal Class II malocclusion was positioned farther back compared with that of patients with skeletal Class III malocclusion. In general, mandibles that are shorter and/or located farther back might push the tongue and soft palate back into the pharyngeal space, thus reducing the oropharyngeal volume. Patients in Class III had a more protruded mandible; thus, the hyoid bone had a more anterior position, accounting for the larger distance between the back of the tongue and the posterior pharyngeal wall. Therefore, patients in Class III had the largest airway volumes. Consistent with the aforementioned reports [14, 16], the current study also found that the horizontal distance of the hyoid bone and Pog among patients in Class II was significantly smaller than that among patients in Class I and Class III.

When the related structural positions of Pog, H, and C2 on the FH plane were evaluated in terms of Or–Po–Pog and Or–Po–C2 angles, the angles of patients in Class II were significantly larger than those of patients in Class I and Class III. No significant difference was observed in the Or–Po–H angle between the three groups; however, the horizontal position of H in patients in Class II was significantly farther back than that in patients in Class I and Class III, indicating that patients in Class II would raise their heads to elevate the FH planes more to compensate for smaller airways, which explains the absence of a significant difference between the Or–Po–H angles of the patients in the three groups. When the airway was examined through the cervical spine and related structural positions through C2, no significant difference was observed in terms of the Po–C2–Pog and Po–C2–H angles of patients in Class I and Class III, reflecting that the pharyngeal airway spaces of those in Class I and III also showed no significant difference. By contrast, the angles of patients in Class II were the smallest, and their airways were also the smallest, probably representing a compensation mechanism for maintaining an airway patency and function when the glossopharyngeal airway volume decreases.

The minimum cross-sectional area is an important factor in the evaluation of the obstruction potency of the pharyngeal airway. Pharyngeal airway obstruction in patients with sleep apnea manifests through not only reduced airway volume but, more crucially, also compressed area (the minimum cross-sectional area). Trudo et al. [17] had shown by state-dependent imaging that the mean minimal cross-sectional airway area was reduced by 228% (*p* = 0.004) in the retropalatal region (UOA) and by 22% (*p* = 0.02) in the retrolingual region (LOA) during sleep in normal subjects. Therefore, both of UOA and LOA collapse partially and cause the changes of airflow dynamic during sleep, especially in UOA. Alves et al. [11] observed significant differences between the minimum cross-sectional areas of the airways of patients in Class I and Class II. Claudino et al. [8] reported that the minimum cross-sectional areas of the lower pharynx, velopharynx, and oropharynx as well as the mean cross-sectional area of patients in Class II were all smaller than those of patients in Class III. The current research revealed that in terms of the minimum cross-sectional area (MUOA, MLOA, and MTOA) of the oropharyngeal airway, no significant difference was observed between patients in Class I and II. However, the minimum cross-sectional area (MUOA, MLOA, and MTOA) of patients in Class III was significantly greater than that of patients in Class II. More importantly, the present study revealed the area with the highest frequency of MTOA during pharyngeal airway obstruction. Two-thirds of the patients in Class I and Class II had an MTOA in the UOV, and approximately 50% of patients in Class III had an MTOA in both the UOV and LOV. This indicates that the position of Pog in patients in Class III could enlarge the MUOA and UOA more than the MLOA and LOA. Therefore, different obstruction areas of the pharyngeal airways were observed in the three skeletal patterns.

Grauer et al. [18] reported that the glossopharyngeal airway volumes of patients in Class II were smaller than those of patients in Class I. This reduction in pharyngeal airway volume was mainly due to the mandible position being farther back. Moreover, Castro-Silva et al. [19] reported that the mean volume of the pharyngeal airway space among patients in Class III was significantly greater than that among patients in Class I and Class II. The current results are consistent with those reported by Castro-Silva et al., [14] in which the oropharyngeal airway volumes of patients in Class III were significantly larger than those of patients in Class II. Contrary to the findings of Grauer et al. [18], those of the present study revealed that no significant difference existed in oropharyngeal airway volume between patients in Class I and Class II.

Analysis using Pearson's correlation coefficient revealed significant correlations between sex and airway space in terms of both oropharyngeal area and oropharyngeal volume; the values for airways were significantly higher in male patients than in female patients, and ANB was significantly negatively correlated with all of the airway spaces. In addition, when the correlation of airway space alone was considered with respect to the three skeletal pattern types, a significant positive correlation was observed in MUOA and MTOA, whereas a significant negative correlation was observed between the ANB and the oropharyngeal airway variables. The positions of the hyoid bone and Pog were nearly significantly correlated with the area or volume of the oropharyngeal airway space, and the correlation strength of PogX (horizontal position) was greater than that of HX (horizontal position). Moreover, the correlation strength of HY (vertical position) was greater than that of PogY (vertical position). From the Or–Po–Pog angles of the head position (FH plane), significant negative correlations were observed with the measurements of all of the oropharyngeal airways; that is, the airway space was smaller when the Pog was farther back. Moreover, when observed from the cervical spine, several negative correlations with Po–C2–C4 were observed, indicating that the angle of the cervical spine affected the volume and area of the oropharyngeal airway space.

## 5. Conclusion

The oropharyngeal airway areas and volumes of patients in Class II were significantly smaller than those of patients in Class III. The positions of the mandible, head, and cervical spine were important factors affecting the oropharyngeal airway area and volume. The minimum oropharyngeal cross-sectional area had a 66%–67% probability of occurrence in the upper oropharyngeal airway among patients in Class I and Class II and a 50% probability of occurrence among patients in Class III.

## Figures and Tables

**Table 1:** Patient's characteristics in the skeletal patterns using one-way ANOVA with Tukey's HSD post hoc test.

Variables	Class I		Class II		Class III				Intergroup comparison
	Mean	SD	Mean	SD	Mean	SD	*F*	*p* value	
Age	25.3	5.70	25.8	5.95	23.8	5.54	1.897	0.154	─
ANB	2.2	1.21	7.1	2.05	-4.4	3.10	279.543	<0.001^∗^	Class II > I > III
BMI	21.9	3.44	20.9	2.87	21.7	3.34	1.152	0.319	─
Hyoid									
Horizontal	33.7	6.18	27.1	5.95	36.1	7.09	24.721	<0.001^∗^	Class III > II, Class I > II
Vertical	74.1	10.37	71.7	8.80	70.8	8.14	1.522	0.222	─
Pogonion									
Horizontal	74.3	7.84	63.7	8.42	80.2	10.43	40.442	<0.001^∗^	Class III > I > II
Vertical	77.5	9.87	74.6	8.74	74.0	8.46	1.684	0.189	─

BMI: body mass index. ^∗^Intergroup comparison: statistically significant, *p* < 0.05.

**Table 2:** The measured angles in the skeletal patterns using one-way ANOVA with Tukey's HSD post hoc test.

Angles	Class I		Class II		Class III				Intergroup comparison
	Mean	SD	Mean	SD	Mean	SD	*F*	*p* value	
Or-Po-Pog	45.6	4.13	48.9	4.29	41.5	3.41	51.371	<0.001^∗^	Class II > I > III
Or-Po-H	65.7	4.70	69.3	4.72	69.1	58.65	0.077	0.926	─
Or-Po-C2	83.8	5.86	87.3	5.15	82.5	4.90	11.318	<0.001^∗^	Class II > I, Class II > III
Po-C2-Pog	113.0	8.78	108.9	6.58	110.6	6.97	2.832	0.062	─
Po-C2-H	130.6	12.54	124.8	12.00	124.8	10.56	3.09	0.049^∗^	Class I > II
Po-C2-C4	192.7	5.44	193.2	7.30	189.4	4.67	7.307	0.001^∗^	Class II > III, Class I > III

Or: orbitale; Po: porion; Pog: pogonion; H: hyoid bone; C2: second cervical vertebra; C4: fourth cervical vertebra. ^∗^Intergroup comparison: statistically significant, *p* < 0.05. ─: not significant.

**Table 3:** Oropharyngeal airway spaces in the skeletal patterns using one-way ANOVA with Tukey's HSD post hoc test.

Angles	Class I		Class II		Class III				Intergroup comparison
	Mean	SD	Mean	SD	Mean	SD	*F*	*p* value	
UOA (mm^2^)	443.9	92.46	377.2	91.29	468.5	121.77	9.342	<0.001^∗^	Class III > II, Class I > II
MUOA (mm^2^)	95.1	45.36	78.8	42.41	118.3	55.94	8.489	<0.001^∗^	Class III > II
UOV (mm^3^)	12682.9	4100.43	10658.7	3425.23	13801.9	5466.62	5.823	0.004^∗^	Class III > II
LOA (mm^2^)	272.8	89.49	225.4	91.94	289.7	111.49	5.237	0.006^∗^	Class III > II
MLOA (mm^2^)	110.6	33.23	86.0	38.79	113.4	51.72	5.117	0.007^∗^	Class III > II
LOV (mm^3^)	7159.7	3020.93	6051.5	3215.32	7773.5	3912.60	3.070	0.049^∗^	Class III > II
MTOA (mm^2^)†	85.5	34.12	69.6	33.83	96.0	42.35	6.200	0.003^∗^	Class III > II
TOV (mm^3^)	19842.6	5952.78	16710.1	6007.16	21575.4	8638.67	5.535	0.005^∗^	Class III > II

UOA: upper orophyngeal area; MUOA: minimum upper oropharyngeal area; UOV: upper oropharyngeal volume; LOA: lower oropharyngeal area; MLOA: minimum lower phsryngeal area; LOV: lower oropharyngeal volume; MTOA: minimum total oropharyngeal area; TOV: total oropharyngeal volume. †MTOA: Class I (20 MUOA + 10 MLOA); Class II (27 MUOA + 13 MLOA); Class III (39 MUOA + 38 MLOA). ^∗^Intergroup comparison: statistically significant, *p* < 0.05. ─: not significant.

**Table 4:** Pearson test of oropharyngeal airway in the patient's characteristics.

	UOA	MUOA	UOV	LOA	MLOA	LOV	MTOA	TOV
Gender	0.253^∗^	0.247^∗^	0.235^∗^	0.400^∗^	0.322^∗^	0.371^∗^	0.247^∗^	0.322^∗^
Skeletal	0.161	0.231^∗^	0.149	0.123	0.072	0.111	0.163^∗^	0.146
Age	-0.149	-0.164^∗^	-0.104	-0.221^∗^	-0.091	-0.150	-0.171^∗^	-0.136
BMI	0.038	-0.030	-0.075	0.118	0.054	-0.007	-0.041	-0.051
ANB	-0.348^∗^	-0.349^∗^	-0.272^∗^	-0.294^∗^	-0.237^∗^	-0.234^∗^	-0.292^∗^	-0.281^∗^
HX	0.386^∗^	0.341^∗^	0.288^∗^	0.232^∗^	0.224^∗^	0.211^∗^	0.265^∗^	0.280^∗^
HY	0.281^∗^	0.237^∗^	0.296^∗^	0.388^∗^	0.198^∗^	0.392^∗^	0.219^∗^	0.370^∗^
PogX	0.476^∗^	0.455^∗^	0.387^∗^	0.333^∗^	0.318^∗^	0.293^∗^	0.384^∗^	0.381^∗^
PogY	0.202^∗^	0.217^∗^	0.229^∗^	0.202^∗^	0.090	0.192^∗^	0.186^∗^	0.234^∗^

UOA: upper oropharyngeal area; MUOA: minimum upper oropharyngeal area; LOA: lower oropahryngeal area; MLOA: minimum lower oropharyngeal area; UOV: upper oropharyngeal volume; LOV: lower oropharyngeal volume; MTOA: minimum total pharyngeal area; TOV: total oropharyngeal volume; BMI: body mass index; HX: hyoid (horizontal); HY: hyoid (vertical); PogX: pogonion (horizontal); PogY: pogonion (vertical). ^∗^Statistically significant, *p* < 0.05.

**Table 5:** Pearson test of measured angles and oropharyngeal airway.

Variables	UOA	MUOA	UOV	LOA	MLOA	LOV	MTOA	TOV
Or-Po-Pog	-0.307^∗^	-0.275^∗^	-0.196^∗^	-0.190^∗^	-0.249^∗^	-0.142	-0.244^∗^	-0.190^∗^
Or-Po-H	0.157	0.202^∗^	0.185^∗^	0.113	0.038	0.168^∗^	0.086	0.195^∗^
Or-Po-C2	-0.099	-0.094	-0.023	-0.002	0.030	0.013	-0.015	-0.009
Po-C2-Pog	-0.021	-0.038	-0.069	-0.113	-0.150	-0.129	-0.086	-0.104
Po-C2-H	0.002	-0.051	-0.030	0.039	-0.082	0.039	-0.055	-0.001
Po-C2-C4	-0.295^∗^	-0.230^∗^	-0.251^∗^	-0.180^∗^	0.153	0.118	-0.182^∗^	-0.213^∗^

UOA: upper oropharyngeal area; MUOA: minimum upper oropharyngeal area; LOA: lower oropharyngeal area; MLOA: minimum lower oropharyngeal area; UOV: upper oropharyngeal volume; LOV: lower oropharyngeal volume; MTOA: minimum total oropharyngeal area; TOV: total oropharyngeal volume; Or: orbitale; Po: porion; Pog: pogonion; H: hyoid bone; C2: second cervical vertebra; C4: fourth cervical vertebra.^∗^Statistically significant, *p* < 0.05.

## Data Availability

The data used to support the findings of this study are included within the article. The data used to support the findings of this study are available from the corresponding author upon request.
